# The Tumor Multi-Omic Landscape of Endometrial Cancers Developed on a Background of Adiposity

**DOI:** 10.3390/genes17070744

**Published:** 2026-06-29

**Authors:** George Richenberg, Amy Francis, Carina N. Owen, Victoria Gray, Timothy Robinson, Aurélie A. G. Gabriel, Kate Lawrenson, Emma J. Davidson, Joellen M. Schildkraut, James D. Mckay, Tom R. Gaunt, Caroline L. Relton, Emma E. Vincent, Siddhartha P. Kar

**Affiliations:** 1 MRC Integrative Epidemiology Unit, University of Bristol, Bristol BS8 1QU, UK; 2 Population Health Sciences, Bristol Medical School, University of Bristol, Bristol BS8 1QU, UK; 3 Cancer Epidemiology and Prevention Research Unit, School of Public Health, Imperial College London, London SW7 2AZ, UK; 4 Bristol Cancer Institute, University Hospitals Bristol and Weston NHS Foundation Trust, Bristol BS1 3NU, UK; 5 Division of Infection and Immunity, Cardiff University School of Medicine, Cardiff CF14 4XN, UK; 6 North Wales Medical School, Bangor University, Bangor LL57 2DG, UK; 7 Genomic Epidemiology Branch, International Agency for Research on Cancer/World Health Organization (IARC/WHO), 69372 Lyon, France; 8 Department of Pathology and Immunology, Faculty of Medicine, University of Geneva, 1205 Geneva, Switzerland; 9 Department of Obstetrics and Gynecology, University of Texas Health Science Center at San Antonio, San Antonio, TX 78229, USA; 10 Division of Cancer Sciences, Faculty of Biology, Medicine and Health, University of Manchester, Manchester M13 9PL, UK; 11 Department of Obstetrics and Gynaecology, St. Mary’s Hospital, Manchester Academic Health Science Centre, Manchester University NHS Foundation Trust, Manchester M13 9WL, UK; 12 Department of Epidemiology, Rollins School of Public Health, Emory University, Atlanta, GA 30322, USA; 13 London School of Hygiene and Tropical Medicine, University of London, London WC1E 7HT, UK; 14 Translational Health Sciences, Bristol Medical School, University of Bristol, Bristol BS8 1QU, UK; 15 The Li Ka Shing Early Cancer Institute, University of Cambridge, Cambridge CB2 0XZ, UK; 16 Centre for Cancer Genetic Epidemiology, University of Cambridge, Cambridge CB1 8RN, UK; 17 Department of Oncology, University of Cambridge, Cambridge CB2 0XZ, UK

**Keywords:** endometrial cancer, adiposity, body mass index, germline, polygenic scores, somatic, multi-omics

## Abstract

**Background**: High body mass index (BMI) is a causal risk factor for endometrial cancer, but the tumor molecular mechanisms affected by adiposity remain poorly understood. Here, we characterize the tumor multi-omic landscape of endometrial cancers that have developed on a background of lifelong germline genetic liability to elevated BMI. **Methods**: We built a polygenic score (PGS) for BMI in women using data on independent, genome-wide significant variants associated with adult BMI in 434,794 women. We performed germline (blood) genotype quality control and imputation on data from 354 endometrial cancer cases from The Cancer Genome Atlas (TCGA). We assigned each case in this TCGA cohort their genetically predicted BMI based on the BMI PGS. Multivariable generalized linear models adjusted for age, stage, microsatellite status and genetic principal components were used to test for associations between the BMI germline PGS and endometrial cancer tumor genome-wide genomic, transcriptomic, proteomic, epigenomic and immune traits in TCGA. **Results**: High BMI germline PGS was associated with (i) upregulated tumor gene expression in *IL6*-*JAK*-*STAT3* signaling (FDR = 4.2 × 10^−7^) and in other immune/inflammatory pathways; (ii) increased estimated intra-tumor activated mast cell infiltration (FDR = 0.008); and (iii) increased single base substitution (SBS) mutational signature 1 (FDR = 0.03), implicating age-related mutagenesis. In contrast, BMI at diagnosis associated with elevated progesterone receptor expression and alterations in estrogen and androgen signaling. **Conclusions**: Thus, we integrated germline, somatic and clinical data to identify associations between genetically predicted lifelong liability to higher BMI and endometrial cancer tumor molecular features. These associations inform our understanding of how high BMI may influence the development of this cancer, shaping endometrial tumor biology differentially over the long term.

## 1. Introduction

Endometrial cancer is the second most common malignancy of the female genital tract and the sixth most common cancer among women worldwide [1]. In 2022, there were over 420,000 new endometrial cancer cases and 97,000 deaths from this cancer globally [1], and its incidence and mortality are rising [2]. Several risk factors are associated with endometrial cancer [3,4,5]. These include higher body mass index (BMI; the most common measure of adiposity) and obesity, long-term unopposed estrogen exposure, insulin resistance and diabetes, and inherited genetics, particularly Lynch syndrome that involves germline mutations in mismatch repair genes. Over 50% of endometrial cancer in the population is attributable to overweight and obesity [6,7]. Meta-analyses of observational studies have shown that the relative risk for endometrial cancer for a 5 kg/m^2^ increase in BMI is approximately 1.5 [8] and those in the highest BMI category have a 7-fold elevated risk of developing this cancer than those with normal BMI [9]. Mendelian randomization (MR) studies [10], which leverage the random allocation at conception of germline genetic variants robustly associated with BMI to proxy lifelong genetic liability for higher BMI, have confirmed that the association between higher BMI and endometrial cancer risk is causal [11,12,13].

Investigating the biological basis of the adiposity–endometrial cancer link has been identified as a top research priority by patients, survivors, carers and clinicians [14]. MR mediation analyses have suggested that only 7 to 19% of the effect of BMI on endometrial cancer risk may be mediated by circulating insulin, testosterone and sex hormone binding globulin levels [11], indicating that most of the effect of BMI occurs via mechanisms that are as yet unexplained. The search for endometrial tissue-specific effects of high BMI has been elusive and focused on the interface between IGF1 receptor signaling and the PI3K/AKT/mTOR pathway in the context of endometrial hyperplasia [15,16,17]. These studies have been limited to using non-human model organisms, and, for human studies, small sample sizes and an emphasis on cell line experiments. Moreover, despite the established association between increased BMI and endometrial cancer, little is known about how long-term adiposity shapes endometrial tumor biology and how this differs from the association between BMI at diagnosis and endometrial tumor biology. Relationships between long-term adiposity and the endometrial tumor molecular profile may reflect key steps in tumor initiation and development and thereby reveal actionable preventative and potentially therapeutic targets. Such relationships have the potential to form the basis of precision oncologic interventions in women with a history of overweight or obesity who develop endometrial cancer.

**Figure 1:**
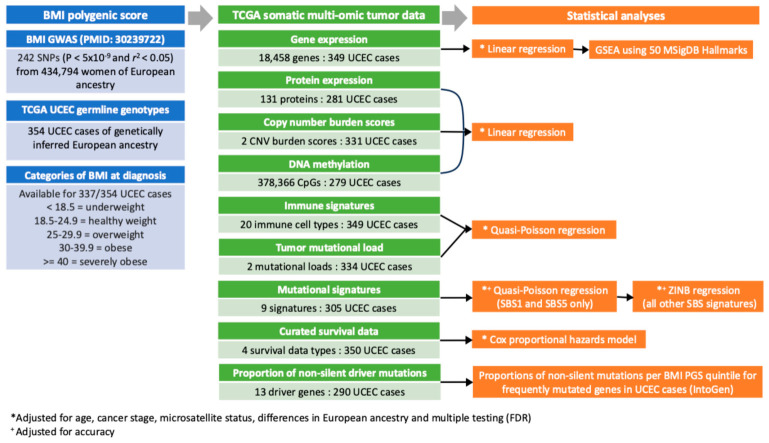
Schematic overview of study design, data sources, and analyses performed.

Here, we apply a unique study design (Figure 1) that uses a polygenic score (PGS) for BMI to evaluate the association between adiposity and endometrial cancer tumor multi-omic traits. BMI PGS, which represents the aggregate effects of multiple BMI-associated germline variants, better captures lifelong liability to adiposity [18,19,20]. In contrast, BMI measured at a single timepoint such as at cancer diagnosis may—at least to some extent—be affected by the developing cancer itself (reverse causation) or confounded by other factors. We exploit The Cancer Genome Atlas (TCGA) Uterine Corpus Endometrial Carcinoma (UCEC) project data that includes tumor genomic, transcriptomic, proteomic and epigenomic profiles of over 350 endometrial cancer tumors with matched blood-based germline genotype data, assigning each participant in this cohort their PGS for BMI [21]. We obtain this BMI PGS from a genome-wide association study (GWAS) of over 434,000 women [18], combining the effects of independent, genome-wide significant single nucleotide polymorphisms (SNPs) associated with BMI. Utilizing this study design integrating BMI GWAS data with the germline and somatic components of the TCGA endometrial cancer cohort enabled us to uncover key associations between genetic liability to elevated BMI and tumor genomic, transcriptomic and proteomic features. These associations may offer potential opportunities for early intervention and targeted treatment in endometrial tumors developing on a background of adiposity.

## 2. Materials and Methods

### 2.1. Germline Genetic Data and BMI Polygenic Score

Blood-derived DNA (germline DNA) from the TCGA UCEC cohort had been genotyped by the TCGA Research Network using the Affymetrix Genome-Wide Human SNP Array 6.0. We obtained genetically inferred ancestry calls for this cohort from Carrot-Zhang et al. based on a consensus of five independent ancestry classification methods [22]. For the cases in the cohort classified as being of genetically inferred European ancestry, we downloaded the genotypes called by the Birdseed algorithm from the controlled tier of the legacy Genomic Data Commons (GDC) portal accessed via an approved application to the database of Genotypes and Phenotypes (dbGAP). Genotypes with Birdseed confidence score ≥ 0.1 were set as missing (based on Affymetrix recommendation) and REF and ALT alleles were aligned to the forward strand. Ambiguous SNPs (SNPs with A/T or C/G alleles) were removed. Genotype calls with an SNP call rate > 95%, minor allele frequency > 0.5%, and Hardy–Weinberg equilibrium exact test *p* ≥ 10^−6^; and samples with >95% call rate, inbreeding coefficient, F such that |F| ≤ 0.2, and kinship coefficient ≤ 0.0884 were retained. These filters were applied using VCFtools (v0.1.16) [23]. Retained genotypes were phased and imputed to the 1000 Genomes Phase 3 (Version 5) reference panel [24] using Eagle2 [25] and Minimac4 [26], respectively.

Data for the BMI PGS were obtained from a GWAS meta-analysis that involved 434,794 adult women of European ancestry from the Genetic Investigation of Anthropometric Traits (GIANT) consortium and the UK Biobank [18]. Specifically, we used effect size estimates (regression beta coefficients) and corresponding allelic information for index variants associated with BMI in women at genome-wide significance (*p* < 5 × 10^−9^) that were independent (*r*^2^ < 0.05) on linkage disequilibrium (LD) clumping performed using a reference panel of 20,275 unrelated individuals from the UK Biobank [18]). Each case in the TCGA UCEC cohort was then assigned their genetically predicted BMI (BMI PGS) using the beta coefficients from the BMI GWAS meta-analysis with PGS calculation implemented using the PRSice-2 software [27]. SNPs with ambiguous alleles were removed and the final BMI PGS was calculated based on 242 SNPs captured in the GWAS and TCGA data. Specifically, for the PGS calculation, the betas for each BMI-associated index SNP were multiplied by the effect allele count for that SNP for each case in the TCGA UCEC cohort. These numbers were then summed over all 242 SNPs to assign each case in the cohort their BMI PGS. The PGS was standardized by subtracting the mean PGS for the cohort and dividing by the standard deviation.

### 2.2. Tumor Multi-Omic Data

An in-depth description, including details of biospecimen collection, laboratory protocols, quality control and bioinformatic processing pipelines, for the TCGA UCEC tumor multi-omic data sets, is available in Levine et al. [21]. Here, we briefly describe the specific data sets that were analyzed in our study. Tumor gene expression in the UCEC cohort was measured using the Illumina Genome Analyzer RNA-Seq platform. The “RNA-Seq by Expectation Maximization” (RSEM) raw count matrix including 20,531 genes was downloaded from the Firehose portal (gdac.broadinstitute.org). We removed the 10% of genes that had the lowest expression levels and subjected the matrix to quantile normalization and inverse normal transformation. Estimates of the relative fractions of 22 immune cell types within the leukocyte compartment of TCGA UCEC tumors were downloaded from the GDC (gdc.cancer.gov/about-data/publications/panimmune) and filtered to only retain the immune cell types with at least 10% prevalence. These were derived from a previously published application of the “cell-type identification by estimating relative subsets of RNA transcripts” (CIBERSORT) algorithm to the UCEC RNA-Seq data [28,29]. CIBERSORT uses a set of immune cell profiles as reference to calculate a baseline matrix of immune cell signatures that can then be applied to tumor samples to determine relative proportions of immune cells in the leukocyte fraction of the tumor. Tumor protein expression in the UCEC cohort was measured using the reverse phase protein array (RPPA) for 131 proteins and the “level 3” replicate-base normalized protein expression matrix was downloaded from the Xena repository (xena.ucsc.edu).

A matrix of 65 single-base substitution (SBS) tumor mutational signatures called from whole-exome sequencing of 9493 tumors in the TCGA pan-cancer cohort was downloaded from the Xena repository and filtered to only retain mutational signatures with at least 10% prevalence [30]. Tumor mutation load estimates (silent mutations per megabase (Mb) and non-silent mutations per Mb) linked to Thorsson et al. [29] were also downloaded from the GDC for the pan-cancer TCGA cohort and subset to UCEC cases. “TCGA Unified Ensemble MC3” gene-level tumor mutation calls were downloaded from the Xena repository. These calls were obtained from a standardized variant calling pipeline involving an ensemble of seven mutation-calling algorithms with artefact filtering and variant annotation applied to the UCEC tumor exome sequencing data set and carried out as part of the Multi-Center Mutation Calling in Multiple Cancers (MC3) project [31]. Genes in each TCGA tumor were subjected to a binary classification as either wild-type (zero) or carrying a non-silent mutation (one). We focused our analysis on 13 established cancer driver genes that were mutated in at least 10% of endometrial cancers nominated as per the IntegrativeOncoGenomics portal (intogen.org). Genome-wide DNA methylation in the UCEC cohort was measured using the Illumina Infinium HumanMethylation450 array. A matrix of the methylation beta values and corresponding array probe information mapped to cytosine–phosphate–guanine (CpG) sites in the human genome were downloaded from Xena. We removed 107,211/485,578 CpG sites with >80% missing data. Copy number variation (CNV) burden scores were downloaded from the GDC and comprised a number of segments altered in the copy number profile for each UCEC sample and fraction of bases deviating from baseline ploidy [29,32].

### 2.3. Statistical Analyses

We used generalized linear models (GLMs) adjusted for age and stage at diagnosis, 10 genetic ancestry principal components, and microsatellite stability status to evaluate the associations between the germline BMI PGS and TCGA UCEC tumor gene expression, immune signatures, protein expression, copy number burden scores, mutational signatures and tumor mutation load estimates. The default GLM was linear regression and outcome variables such as gene and protein expression were treated as continuous variables with no cut-off applied. For immune and mutational signature and mutational load data, we used quasi-Poisson GLMs. For mutational signatures with >70% zeros, we fit zero-inflated negative binomial (ZINB) models. The GLMs for mutational signatures were also adjusted for signature accuracy. Microsatellite stability was coded as a binary variable (microsatellite stable (MSS) versus instability (MSI)). Age, stage, and MSS/MSI covariates were obtained from the clinical phenotypes matrix file downloaded from Xena while principal components were obtained from Carrot-Zhang et al. [22].

In addition to examining gene expression associations at the level of single genes, we also performed pathway analysis. We ranked the 18,458 genes evaluated genome-wide in the BMI PGS-tumor gene expression analysis by descending order of their expression-association with the BMI PGS using the absolute value of the linear regression Z-score as the ranking metric. This ranked list was then subjected to gene set enrichment analysis (GSEA; [33]) to identify biological pathways enriched among genes whose tumor expression was strongly associated with the BMI PGS. The Molecular Signatures Database Hallmark gene set collection [34] comprising 50 pathways was used for GSEA [34]. GSEA was implemented using fgsea (version 1.26.0) and msigdb (version 7.5.1) R packages. GSEA results were ranked in ascending order of the adjusted *p*-value (i.e., false discovery rate (FDR)). We also conducted an epigenome-wide association study (EWAS) using the CpGassoc R package (version 2.6; [35]) to test for associations between the germline BMI PGS and tumor DNA methylation in the UCEC cohort. The fixed-effects EWAS models were adjusted for the same covariates as the other GLMs described above. Finally, we examined associations between BMI PGS and four survival endpoints in the UCEC cohort: overall survival (OS), disease-specific survival (DSS), progression-free interval (PFI) and disease-free interval (DFI). The definitions and curation of these survival endpoints have been described previously in detail [36] and we downloaded the survival data from the Xena repository. Survival analyses were performed using Cox proportional-hazards models adjusted for age, stage, MSS/MSI status, and 10 genetic principal components and implemented using the survival (version 3.3.1) R package. For the top pathway-level gene expression and protein expression associations with BMI PGS, we used the cBioPortal (PanCancer Atlas version of TCGA) to evaluate associations with OS, PFI, DSS and DFI using Kaplan–Meier plots and log-rank tests [37]. We note that these prognostic associations were independent of the BMI PGS and that the cBioPortal PanCancer Atlas version of the TCGA UCEC cohort was larger than the version of the TCGA UCEC cohort with BMI PGS available. For gene expression, we used the option “mRNA expression z-scores relative to all samples (log RNA Seq V2 RSEM)”, and for protein expression, we used the option “Protein expression z-scores (RPPA)” with the z-score threshold set to the default value of ±2.0.

FDR control was used to account for multiple comparisons in each analysis described above and results at FDR < 0.05 emphasized. To avoid over-reliance on a hard FDR threshold to guide our interpretation, we also explored results achieving FDR < 0.10 [38].

## 3. Results

### 3.1. Correlation of BMI PGS and BMI Measured at Diagnosis

Of the 354 TCGA UCEC cases with matched tumor and germline data who were assigned a BMI PGS, height and weight at diagnosis were available and used to calculate the BMI at diagnosis for 337 patients. After removing 15 cases with extreme BMI values at diagnosis (BMI < 15 kg/m^2^ or >50 kg/m^2^), the BMI PGS, which reflects lifelong genetic liability for higher BMI, was correlated (Pearson’s *r* = 0.23; *p* = 2.1 × 10^−5^; Figure S1A) with the actual measured BMI at the timepoint of diagnosis (median BMI at diagnosis = 31.9 kg/m^2^, interquartile range (IQR) = 12.9 kg/m^2^). BMI at diagnosis is likely to be affected to a certain degree by the prior onset of the cancer itself and is a single-timepoint measure, so we did not expect to see a perfect correlation with the BMI PGS nor complete independence either and the correlation of 0.23 reflects this trade-off. We also used linear regression to test for associations between the BMI PGS and age (median age = 64 years, IQR = 16 years) or stage (stages I/II/III/IV: 67%/8%/21%/4%) at diagnosis or tumor microsatellite status (MSS = 58%, rest MSI) but found no statistically significant associations with these variables (Figure S1B–D). There was no visible patterning of the endometrial cancer histological and molecular subtypes by increasing BMI quintiles (Figure S2A,B).

### 3.2. Associations Between BMI PGS and Tumor Gene Expression

**Figure 2:**
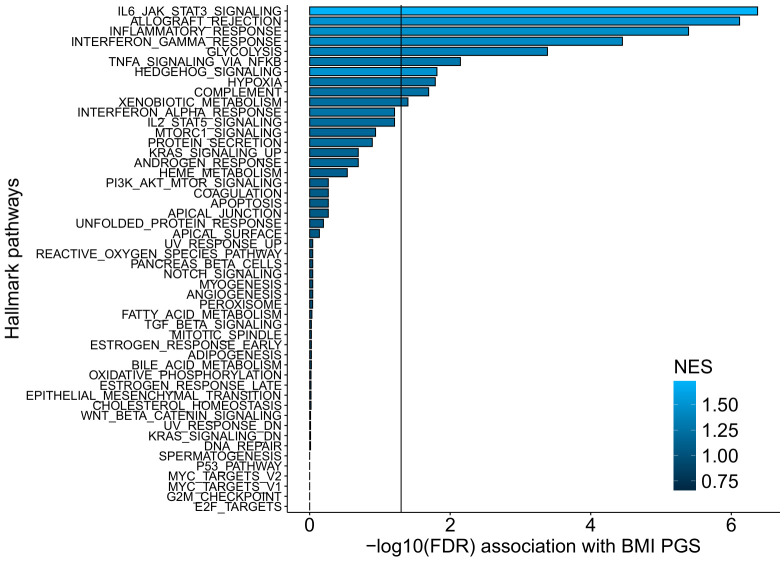
Gene set enrichment analysis (GSEA) results for 50 Hallmark pathways for the association between BMI PGS and tumor gene expression (RNA-Seq) in the TCGA UCEC cohort. The strength of enrichment associations corrected for multiple comparisons (−log_10_(FDR)) for each of the 50 pathway gene sets evaluated is shown. The vertical line indicates FDR = 0.05 and the histogram bars are colored based on GSEA normalized enrichment scores (NES) for each pathway.

**Figure 3:**
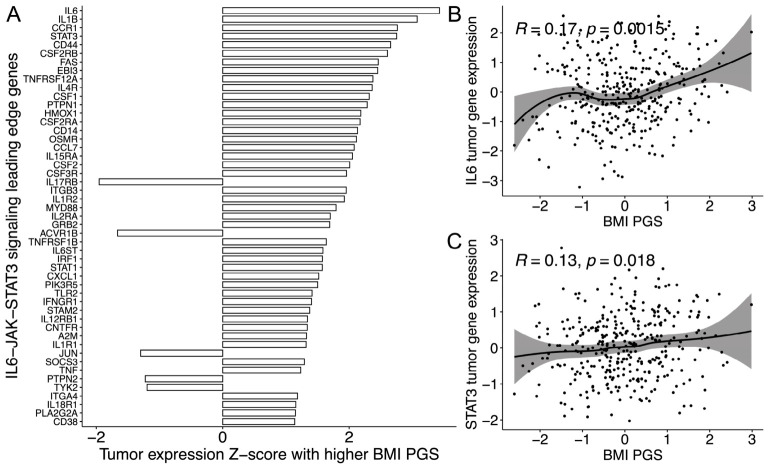
The direction of association (regulation) between BMI PGS and TCGA UCEC tumor expression of specific genes in the IL6-JAK-STAT3 signaling Hallmark pathway. (**A**) Z-scores with respect to higher BMI PGS for each of the 49 leading edge genes driving the IL6-JAK-STAT3 signaling pathway association in GSEA. Each bar represents the gene’s Z-score for its association with the BMI PGS, where positive values indicate that gene expression increases as BMI PGS increases and negative values indicate that gene expression decreases as BMI PGS increases. The Z-score is the beta coefficient (effect size) divided by the standard error (measure of uncertainty) from linear regression of gene expression on BMI PGS adjusted for age, stage, genetic ancestry, and microsatellite status. Scatter plot and LOESS (locally weighted smoothing) line with 95% confidence interval shaded displaying the association between BMI PGS and inverse normal rank transformed tumor gene expression for (**B**) *IL6* and (**C**) *STAT3*. The Pearson correlation coefficient is shown while linear regression *p*-values adjusted for age, stage, microsatellite status, and 10 genetic principal components are provided in Table S3.

We tested for associations between the BMI germline PGS and the tumor RNA-Seq-based expression level of 18,458 genes across the genome in 349 TCGA UCEC cases with matched germline DNA-somatic RNA-Seq data. None of the single gene transcriptomic associations achieved FDR < 0.05 after correcting for multiple comparisons (Table S1). However, ranking the genes in descending order of their strength of tumor transcriptomic association with the BMI PGS and performing GSEA using the 50 Hallmark gene set biological pathways identified profound pathway-level enrichment, with tumor gene expression changes in 11 pathways associated with the BMI PGS at FDR < 0.05 (Table S2). The top pathway association with the BMI PGS was IL6-JAK-STAT3 signaling tumor expression (*p* = 8.5 × 10^−9^; FDR = 4.2 × 10^−7^; Figure 2; Table S2). A total of 49 of the 86 genes in the IL6-JAK-STAT3 pathway were defined by GSEA as “leading edge” genes (i.e., responsible for driving the enrichment signal) and 44 of these 49 genes were upregulated with increasing BMI PGS (Figure 3A; Table S3). These included both key drivers *IL6* (the gene most strongly upregulated with higher BMI PGS in this pathway; *p* = 6.8 × 10^−4^; Figure 3B; Table S3) and *STAT3* (the fourth most strongly upregulated gene; *p* = 6.3 × 10^−3^; Figure 3C; Table S3). The other pathways with FDR < 0.05 highlighted a range of inflammatory and immune-mediated processes including allograft rejection and inflammatory and interferon gamma response pathways (Figure 2; Table S2).

### 3.3. Associations Between BMI PGS and Tumor Immune Signatures

**Figure 4:**
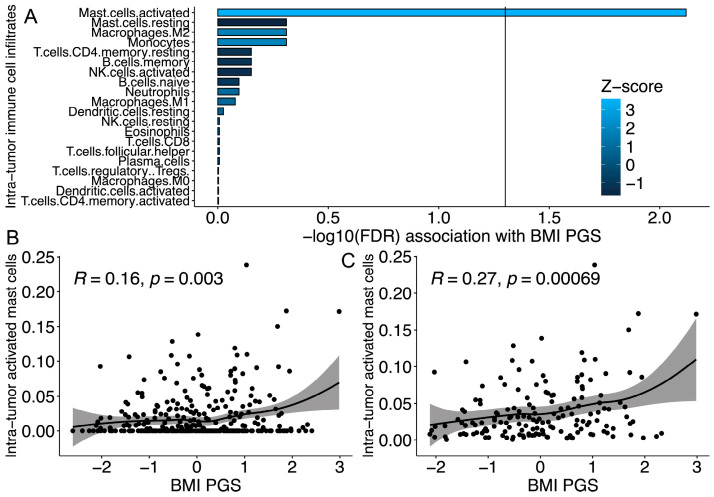
Associations between the BMI PGS and intra-tumor immune cell infiltration levels in the TCGA UCEC cohort. Y-axes for all sub-figures (**A**–**C**) are CIBERSORT-estimated relative immune fractions. (**A**) Associations (−log_10_(FDR)) between the BMI PGS and the CIBERSORT-estimated intra-tumor infiltration levels for 20 immune cell types. The vertical line indicates FDR = 0.05. Scatter plot and LOESS (locally weighted smoothing) line with 95% confidence interval shaded displaying the association between BMI PGS and estimated intra-tumor activated mast cell infiltration levels in (**B**) the full TCGA UCEC cohort (N = 353), which yields the primary estimate, and (**C**) the TCGA UCEC cohort after removing tumors with estimated activated mast cell infiltrate levels of zero (N = 161), which represents an exploratory analysis. The Pearson correlation coefficient is shown while linear regression *p*-values adjusted for age, stage, microsatellite status, and 10 genetic principal components are provided in Table S4.

We examined associations between the BMI PGS and tumor CIBERSORT-based immune signatures that represented estimated intra-tumor infiltration levels of 20 immune cell types in the 349 TCGA UCEC cases with matched germline DNA-somatic RNA-Seq data. We found that higher BMI PGS was strongly associated with elevated levels of activated mast cell intra-tumor infiltration (Z-score = 3.59; *p* = 3.8 × 10^−4^; FDR = 0.008; Figure 4A–C; Table S4).

### 3.4. Associations Between BMI PGS and Tumor Protein Expression

We evaluated associations between the BMI PGS and tumor RPPA-based protein expression levels in 281 TCGA UCEC cases with matched germline DNA-somatic RPPA data. The strongest association was observed for epidermal growth factor receptor (EGFR) expression, assayed by the EGFRPY1173 antibody, which was inversely associated with the BMI PGS (Z-score = −3.50; *p* = 5.6 × 10^−4^; FDR = 0.07; Table S5).

### 3.5. Associations Between BMI PGS and Tumor Mutation Data

In descriptive comparisons, we assessed the proportions of non-silent mutations by BMI PGS quintile for 13 somatic driver genes known to be frequently mutated in endometrial tumors using the 290 TCGA UCEC cases with matched germline DNA-somatic gene-level mutation data (*ARID1A*, *CHD4*, *CTCF*, *CTNNB1*, *FBXW7*, *KMT2D*, *KRAS*, *PIK3CA*, *PIK3R1*, *PPP2R1A*, *PTEN*, *TP53* and *ZFHX3*). We did not observe any clear patterns of increase or decrease in the proportion of mutations in each of these driver genes by progressively increasing quintiles of the BMI PGS in these descriptive comparisons.

Of the 65 single base substitution mutational signatures profiled in TCGA, SBS1 and SBS5 were observed with non-zero values in at least 94% of the UCEC cohort while SBS2, SBS10A, SBS10B, SBS13, SBS15, SBS40 and SBS44 had non-zero prevalence in 10–28% of the cohort. Elevated BMI germline PGS was associated with higher levels of tumor SBS1 (Z-score = 2.97; *p* = 0.003; FDR = 0.027) and showed suggestive associations (FDR < 0.10 but >0.05) with higher SBS5 (Z-score = 2.36; *p* = 0.019; FDR = 0.086) and lower SBS13 (Z-score = −2.16; *p* = 0.031; FDR = 0.093) across 305 UCEC cases with matched germline DNA-somatic mutational signature data (Table S6). We found little evidence of association between the BMI PGS and levels of silent (Z-score = 1.29; *p* = 0.20) and non-silent (Z-score = 1.13; *p* = 0.26) mutations per megabase in 334 UCEC cases with matched germline DNA-tumor mutational load data.

### 3.6. Associations Between BMI PGS and Tumor Copy Number Burden Scores, Methylation, and Survival

**Figure 5:**
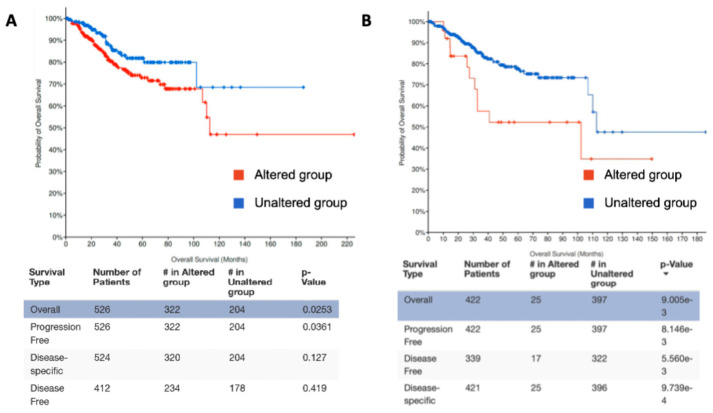
BMI PGS-associated tumor gene and protein expression features and their relationship with survival after a diagnosis of endometrial cancer in the TCGA UCEC cohort. These are prognostic associations at the expression level in the larger cBioPortal PanCancer Atlas version of the TCGA UCEC cohort (overall n = 526 and 422, versus overall n = 354 for the BMI PGS-based analyses) and are independent of the germline BMI PGS. (**A**) IL6-JAK-STAT3 pathway mRNA expression and overall survival, progression-free interval, disease-specific survival, and disease-free interval; with the Kaplan–Meier plot shown for overall survival and (**B**) EGFR phosphorylation/activation and overall survival, progression-free interval, disease-specific survival, and disease-free interval; with the Kaplan–Meier plot shown for overall survival. *p*-values are from log-rank tests comparing TCGA UCEC cases with altered versus unaltered tumor gene or protein expression. # refers to sample size numbers. Altered expression was defined as a Z-score more extreme than ±2.0. The Z-score for each case for a given gene/protein was computed by subtracting the mean expression calculated over the entire TCGA UCEC cohort from the expression in that case and dividing by the cohort-level standard deviation. IL6- JAK-STAT3 pathway membership for the survival analysis comprised 44 genes that were upregulated with higher BMI and drove the BMI PGS GSEA association for this pathway (i.e., the “leading edge” genes; Figure 3).

We found no evidence of associations between the BMI PGS and the number of segments (Z-score = 0.14; *p* = 0.89) and fraction of genome (Z-score = −1.15; *p* = 0.25) subject to copy number alterations in the 331 UCEC cases with matched germline DNA-somatic copy number data. We also conducted an EWAS to identify associations between the BMI PGS and tumor DNA methylation levels across 378,366 genome-wide CpG sites in 279 UCEC cases with matched germline DNA-somatic methylation. None of the CpG sites reached epigenome-wide significance (*p* < 1.3 × 10^−7^; Table S7). We did not identify any statistically significant associations between the BMI PGS and the four survival endpoints (OS, DSS, DFI, PFI) in the 350 UCEC cases with matched PGS and survival data (Table S8). However, aberrant expression in the UCEC cohort of the 44 upregulated “leading edge” genes responsible for the IL6-JAK-STAT3 pathway-BMI PGS association was in turn associated with inferior survival (P_OS_ = 0.03 and P_PFI_ = 0.04, respectively; Figure 5A). Altered EGFR protein expression (assayed by the EGFRpY1173 antibody) was also associated with poor survival (P_OS_ = 9.0 × 10^−3^ and P_PFI_ = 8.2 × 10^−3^; Figure 5B).

### 3.7. Associations Between BMI Measured at Endometrial Cancer Diagnosis and Tumor Molecular Features

We also evaluated associations between actual measured BMI at endometrial cancer diagnosis divided into the standard clinical categories of underweight, healthy weight, overweight, obese, and severely obese (Figure 1; we combined underweight with healthy weight given that only a few cases were underweight) and tumor gene and protein expression, and mutational signatures in the TCGA UCEC cohort (Tables S9–S13). Higher BMI at diagnosis was associated with substantial changes in gene expression (449 genes at FDR < 0.05 and 829 genes at FDR < 0.10; Table S9) with the top association emerging as elevated *PGR* (which encodes the progesterone receptor, PR) expression, an association that was also captured at the protein level (Table S12). Gene expression alterations were enriched in six pathways at FDR < 0.05 (Table S10), including in early estrogen and androgen response signaling genes (FDR < 0.05; Table S10). Immunological associations of higher BMI at diagnosis included predicted depletion of activated and elevation of resting dendritic cells (FDR < 0.05; Table S11) and with higher predicted regulatory T cell infiltrate levels (FDR < 0.10; Table S11). Finally, UCEC tumors in cases with higher BMI at diagnosis had lower levels of SBS1 and SBS5 (Table S13), in contrast to the mutational signature profile of tumors with a background of high BMI PGS.

## 4. Discussion

Increased BMI is a causal risk factor for endometrial cancer. Despite this established association, the underlying molecular mechanisms and tissue-level pathways that link higher BMI to endometrial cancer development and the impact of long-term adiposity on endometrial tumor biology remain poorly understood. Against this background, here, we adopt a unique approach examining associations between a germline PGS for BMI and tumor multi-omic data for endometrial cancer, performing the largest and most comprehensive investigation of the effects of adiposity on endometrial cancer molecular profiles. The PGS represents the lifelong genetic liability for higher BMI that is more likely to capture the true long-term effects of adiposity on endometrial cancer initiation, development, and biology than BMI measured at any single time point such as on cancer diagnosis. Our study design was enabled by the availability of PGS from large-scale GWAS of adult BMI in women and blood-based germline genotype data matched to endometrial cancer tumor multi-omic data from TCGA.

We identified associations at FDR < 0.05 between germline genetically predicted BMI and (i) tumor gene expression changes in multiple inflammatory and immune pathways, especially IL6-JAK-STAT3 signaling, (ii) intra-tumor mast cell infiltration, and (iii) single base substitution mutational signature 1. We also show that the germline BMI PGS is not associated with survival after a diagnosis of endometrial cancer. Although the BMI PGS was built from variants robustly associated with BMI in a large GWAS and is an established proxy for long-term adiposity exposure, some BMI-associated variants may also influence metabolic, endocrine, and inflammatory pathways that could affect endometrial tumor biology independently of adiposity. Therefore, while our findings are most consistent with the biological consequences of lifelong genetic liability to higher BMI, we cannot completely exclude the possibility that some associations reflect pleiotropic effects of BMI-associated variants.

When we examined the association between germline genetically predicted BMI and tumor gene expression by placing them in the powerful biological context of well-defined molecular pathways, we identified associations between 11 pathways and genetically predicted BMI at FDR < 0.05, with the IL-6-JAK-STAT3 pathway yielding by far the strongest association in terms of FDR. This suggests that BMI has modest but statistically significant effects on tumor transcription in endometrial cancers in a coordinated manner within these biological pathways. Notably, none of the single gene-level expression associations remained significant after correction for genome-wide multiple testing. The pathway-level findings therefore appear to be driven by the cumulative effect of modest but coordinated expression changes across multiple genes within well-defined biological pathways, a pattern that GSEA is specifically designed to detect. These results support the presence of moderate pathway-wide transcriptional perturbation associated with genetic liability to higher BMI rather than the impact of a small number of individually significant genes. We observed moderate but widespread upregulation of genes involved in IL-6-JAK-STAT3 signaling, with *IL6* and *STAT3* ranking among the top four gene-level associations in this pathway. Obesity is increasingly being recognized as an activator of chronic JAK-STAT signaling mediated via cytokines such as IL6 [39]. Aberrant hyperactivation of the IL6-JAK-STAT3 pathway is seen in several cancer types and associated with an inferior prognosis [40], and we found that that altered expression of genes in this pathway was also associated with worse overall survival in TCGA UCEC, albeit in the larger PanCancer Atlas version of this cohort, which was independent of the BMI PGS and represents an expression-level prognostic association. These associations were driven by overall survival rather than disease-specific or disease-free survival and may reflect non-cancer mortality (e.g., deaths due to cardiometabolic disease) rather than mortality from endometrial cancer. Previous work in endometrial cancer cellular and mouse models has suggested that adipose-derived stem cells in the tumor microenvironment secrete IL6, which in turn activates its major downstream effector STAT3, and the two collectively promote endometrial cancer proliferation, invasion, and metastasis [41]. Antibodies targeting the IL6 receptor and small-molecular inhibitors of JAK1 and STAT3 have been shown to inhibit endometrial cancer growth both in vitro in cell lines and in vivo in mice [42]. The published literature also provides clues potentially implicating the obesity-driven pro-inflammatory interferon response in endometrial cancer pathogenesis, and in keeping with this, we found that the other top ranked pathways significantly upregulated with higher genetic BMI in our analyses included the inflammatory response and interferon-gamma and -alpha pathways [43,44].

We evaluated the association between germline genetically predicted BMI and the tumor infiltration levels of 20 immune cell types in the TCGA endometrial cancer cohort, identifying a significant association between higher BMI and elevated mast cell infiltrates. These infiltration levels were estimated using the CIBERSORT algorithm based on the tumor expression of genes that define these immune cell types, which has been shown to be a reasonable proxy for actual microscopically measured infiltration [45]. Indeed, a previously published study involving microscopic analysis of 35 immunohistochemically stained surgically resected endometrial adenocarcinoma samples found that tumors with increased mast cell counts displayed greater myometrial invasion [46]. Mast cell infiltration has been reported as an early feature in the development of various cancer types and interestingly, mast cell release of IL-6 and other related angiogenic factors has been shown to have a vital role in neovascularization within the tumor microenvironment [47,48]. Tumor infiltrating mast cells are also known to be associated with resistance to anti-PD1 immunotherapy, which is increasingly being used to treat endometrial cancer [49]. Activated mast cells express the proto-oncogene c-KIT, which encodes the target of tyrosine kinase inhibitors such as imatinib [48]. Moreover, in a small series of 45 patients with endometrial adenocarcinoma, most tumors were found to express kinases that can be targeted by imatinib [50]. However, we do urge caution in the interpretation of our CIBERSORT results given that (i) it is a surrogate for tumor immune cell infiltration based on RNA-Seq data and (ii) mast cells are a relatively rare immune cell type whose levels may not be estimated accurately.

We identified a significant association between germline genetically predicted increased BMI and increased burden of single base substitution mutational signature SBS1 (FDR < 0.05), and suggestive associations (FDR < 0.10 but >0.05) with increased SBS5 and decreased SBS13. Mutational signatures represent the genomic footprints of the impact of various exogeneous and endogenous cancer risk factors on the somatic or tumor tissues but the precise causes for most of the mutational signatures remain ill-defined [51]. SBS1 is characterized by C > T transitions at methylated CpG sites due to spontaneous deamination of 5-methylcytosine, DNA polymerase errors or enzymatic deamination. It is a “clock-like” mutational signature, but it is as yet unclear whether this clock represents time (aging) or the number of cell divisions. The exact molecular processes underlying SBS5 are unknown though it is also regarded as a “clock-like” signature correlated with age but not with cell division rate. SBS5 has been associated with chronic inflammatory states including inflammatory bowel disease and psoriasis [52,53] and the suggestive association that we have uncovered between adiposity and SBS5 in endometrial cancer might reflect the consequence of an adiposity-driven low-grade chronic inflammatory state. Interestingly, SBS1 and SBS5 (along with SBS18, which we could not evaluate due to inadequate numbers) are the only SBS signatures found in normal endometrial epithelial cells [54]. Phylogenetic modeling indicated that the processes contributing to these signatures in normal endometrium were likely to remain active throughout life. This is consistent with recent multivariable Mendelian randomization analyses that have demonstrated that although adult adiposity has a greater impact on endometrial cancer risk, both childhood and adult adiposity remain associated with endometrial cancer risk [55,56]. Thus, placing our findings in the context of what is already known does suggest that obesity may well be a potential driver of SBS1 contributing to the development of endometrial cancer.

We observed a suggestive inverse association (FDR < 0.10) between germline genetically predicted BMI and endometrial cancer tumor phosphorylated EGFR (EGFRpY1173), indicating lower EGFR activation in tumors arising in those with higher BMI genetic liability in TCGA UCEC. Because the RPPA assay measures phosphorylation at tyrosine 1173 rather than total EGFR abundance, this finding reflects receptor activation rather than protein expression. Although dysregulated EGFR signaling has been implicated in the pathogenesis of endometrial cancer [57], our findings should be interpreted cautiously because the association did not reach the conventional FDR < 0.05 threshold and requires independent validation. Future studies should determine whether differences in EGFR activation contribute to biological differences between endometrial cancers arising in different adiposity backgrounds and whether these observations have any therapeutic relevance.

In contrast to the BMI PGS analyses, BMI measured at endometrial cancer diagnosis was most strongly associated with tumor hormonal signaling, including elevated progesterone receptor expression at both the transcript and protein levels and enrichment of estrogen and androgen response pathways. These findings suggest that contemporaneous BMI may capture endocrine or metabolic states closer to diagnosis, whereas germline genetically predicted BMI may better reflect longer-term adiposity-related inflammatory, immune, and mutational processes involved in tumor development. The divergent SBS1 and SBS5 associations, which were increased with BMI PGS but decreased with BMI at diagnosis, further support the interpretation that single-timepoint BMI and genetically predicted lifelong liability to higher BMI are not interchangeable measures of adiposity exposure. However, this contrast should be interpreted cautiously given that the BMI PGS to SBS5 association was only suggestive (FDR < 0.10 but >0.05). The correlation between BMI PGS and BMI measured at diagnosis in the TCGA cohort was modest (*r* = 0.23), suggesting that these measures capture overlapping but substantially distinct dimensions of adiposity exposure. More broadly, these results highlight the value of comparing genetic and measured adiposity metrics to distinguish molecular features linked to long-term cancer development from those associated with the clinical state at diagnosis. One possible explanation is that the BMI PGS captures cumulative biological exposures operating over decades before cancer diagnosis, whereas measured BMI primarily reflects the state at a single point in time. This distinction suggests that obesity-associated endometrial carcinogenesis may involve both long-term inflammatory, immune, and mutational processes that contribute to tumor development and shorter-term hormonal effects that shape tumor characteristics closer to clinical presentation. However, given that SBS1 and SBS5 accumulate over time and with cell division, differences in exposure timing alone are unlikely to fully explain the opposing directions. Notably, the measured BMI GSEA identified enrichment of proliferation-related pathways, including MYC and E2F targets, in addition to hormonal signaling. This raises the possibility that differences in tumor proliferation or molecular subtype composition may influence the observed mutational signature profiles. Furthermore, although our analyses adjusted for age, stage, and microsatellite status, residual differences in tumor characteristics or other unmeasured factors may also contribute to these findings. Finally, while the BMI PGS primarily serves as a proxy for adiposity, some BMI-associated genetic variants may exert pleiotropic effects that are partly independent of adiposity.

Our study has some limitations. First, our analyses were restricted to individuals of European ancestry, and it is unclear whether our findings are generalizable to other populations. The restriction was enforced by the fact that most women in the TCGA UCEC and the BMI GWAS cohorts were of predominantly European genetic ancestry. Second, while we report several statistically robust associations between germline genetically predicted BMI and tumor multi-omic traits in the TCGA UCEC cohort, we note that there is an acute paucity of endometrial cancer data sets with matched germline genotype and multi-omic tumor phenotyping of comparable depth, breadth, and size to TCGA. The generation of additional and ancestrally diverse endometrial cancer data sets with matched germline and somatic profiles is warranted to further validate our findings.

Third, unopposed estrogen signaling is a major risk factor for endometrial cancer and we did not attempt to jointly model the interaction between BMI and lifelong genetically predicted estrogen levels in our study design [58]. Given the novel study design implemented here, investigating associations between a germline genetically predicted risk factor (BMI) and tumor molecular features, including a second risk factor in a multivariable or factorial setting, would have made interpretation more complex. In sum, we cannot rule out the possibility that some of the BMI-tumor trait associations presented here are mediated via estrogen or other hormones such as insulin but that should not detract from the fact that these are ultimately driven by adiposity. Fourth, associations from case-only analyses such as ours are susceptible to collider bias because all individuals included in TCGA UCEC were already cases of endometrial cancer. Since BMI is a strong causal risk factor for endometrial cancer, conditioning on case status can induce associations between BMI genetic liability and other causes of endometrial cancer, or tumor molecular features related to those causes, even if such associations are absent in the source population. This bias cannot be fully removed by statistical adjustment for covariates within a case-only design such as ours, and estimating its magnitude would require external data on the broader set of factors influencing endometrial cancer incidence as well as tumor molecular traits. Ideally, our case-only findings should be replicated in a pre-cancerous endometrial tissue setting to ensure that these associations are operating over the longitudinal process of tumor development. However, such longitudinal normal or pre-cancer tissue collections with germline and multi-omic profiling are not currently available to our knowledge and addressing their lack has been highlighted as a priority for the field [59].

Fifth, sample sizes for the different analyses presented here ranged from 279 to 354 as is routine for studies based on TCGA due to each omic data layer having different sample sizes. For some of these analyses, particularly gene expression and methylation, our study was underpowered for discovery of individual features such as single gene (no gene at FDR < 0.05) and single CpG (no epigenome-wide significant CpG) associations. While statistically, these associations are null, which is to be expected given the modest sample size, these may nevertheless be biologically informative but must be interpreted with caution.

## 5. Conclusions

In conclusion, we identified associations between BMI predicted by germline genetic variants and endometrial cancer tumor gene expression changes in several immune and inflammatory pathways, most notably the IL6-JAK-STAT3 pathway; tumor mast cell infiltration estimates; and mutational signature SBS1. Though requiring replication in independent endometrial cancer and pre-cancer cohorts with integrated germline-somatic data, these associations offer fresh and potential clinically actionable insights into the impact of adiposity on endometrial cancer biology.

## Data Availability

All data sets analyzed in the manuscript are available at https://www.cancer.gov/tcga (accessed on 9 September 2023), https://gdac.broadinstitute.org/ (accessed on 9 September 2023), https://portal.gdc.cancer.gov/ (accessed on 9 September 2023), https://gdc.cancer.gov/about-data/publications/panimmune (accessed on 9 September 2023), https://xena.ucsc.edu/ (accessed on 9 September 2023), and https://github.com/lindgrengroup/fatdistnGWAS (accessed on 9 September 2023). The TCGA germline genotype data set is available on application at https://dbgap.ncbi.nlm.nih.gov (accessed on 9 September 2023) via accession number phs000178.v11.p8.

## Supplementary Materials

The following supporting information can be downloaded at: https://www.mdpi.com/article/10.3390/genes17070744/s1, Figure S1: Associations between the BMI germline PGS and (A) BMI measured at diagnosis (Pearson correlation coefficient; N = 322), (B) age at diagnosis (Pearson correlation coefficient; N = 354), (C) stage at diagnosis (N = 354; simple linear regression beta = 0.05/*p* = 0.39), and (D) tumor microsatellite status (N = 351; simple linear regression beta = 0.04/*p* = 0.72). The scatter plots in (A) and (B) include the LOESS (locally weighted smoothing) line with 95% confidence interval shaded; Figure S2: Distribution of endometrial cancer histological (A) and TCGA molecular (B) subtypes by BMI germline PGS quintiles in the TCGA UCEC cohort; Table S1: Associations between BMI germline PGS and TCGA UCEC tumor gene expression; Table S2: Hallmark gene set enrichment analysis results for BMI germline PGS-TCGA UCEC tumor gene expression associations; Table S3: Results for the 49 “leading edge” genes driving the association between BMI germline PGS and TCGA UCEC tumor IL6-JAK-STAT3 pathway expression; Table S4: Associations between BMI germline PGS and TCGA UCEC intra-tumor immune cell infiltration estimates; Table S5: Associations between BMI germline PGS and TCGA UCEC tumor protein expression; Table S6: Associations between BMI germline PGS and TCGA UCEC SBS tumor mutational signatures; Table S7: EWAS results for CpG sites achieving *p* < 0.001 for association with BMI germline PGS in the TCGA UCEC cohort; Table S8: Associations between the BMI PGS and survival endpoints in the TCGA UCEC cohort; Table S9: Associations between BMI categories at diagnosis and TCGA UCEC tumor gene expression; Table S10: Hallmark gene set enrichment analysis results for BMI categories at diagnosis-TCGA UCEC tumor gene expression associations; Table S11: Associations between BMI categories at diagnosis and TCGA UCEC intra-tumor immune cell infiltration estimates; Table S12: Associations between BMI categories at diagnosis and TCGA UCEC tumor protein expression. Table S13: Associations between BMI categories at diagnosis and TCGA UCEC SBS tumor mutational signatures

## Abbreviations

The following abbreviations are used in this manuscript:

BMI	Body Mass Index
CIBERSORT	Cell-type Identification by Estimating Relative Subsets of RNA Transcripts
CNV	Copy Number Variation
CpG	Cytosine–Phosphate–Guanine
dbGaP	Database of Genotypes and Phenotypes
DFI	Disease-Free Interval
DSS	Disease-Specific Survival
EGFR	Epidermal Growth Factor Receptor
EWAS	Epigenome-Wide Association Study
FDR	False Discovery Rate
FGSEA	Fast Gene Set Enrichment Analysis
GDC	Genomic Data Commons
GIANT	Genetic Investigation of Anthropometric Traits
GLM	Generalized Linear Model
GSEA	Gene Set Enrichment Analysis
GWAS	Genome-Wide Association Study
IGF1	Insulin-like Growth Factor 1
IL6-JAK-STAT3	Interleukin-6/Janus Kinase/Signal Transducer and Activator of Transcription 3
LD	Linkage Disequilibrium
LOESS	Locally Estimated Scatterplot Smoothing
MC3	Multi-Center Mutation Calling in Multiple Cancers
Mb	Megabase
MR	Mendelian Randomization
MSI	Microsatellite Instability
MSigDB	Molecular Signatures Database
MSS	Microsatellite Stable
mTOR	Mechanistic Target of Rapamycin
OS	Overall Survival
PFI	Progression-Free Interval
PGS	Polygenic Score
PI3K	Phosphoinositide 3-Kinase
PI3K/AKT/mTOR	Phosphoinositide 3-Kinase/Protein Kinase B/Mammalian Target of Rapamycin
RNA-seq	RNA Sequencing
RPPA	Reverse Phase Protein Array
RSEM	RNA-Seq by Expectation Maximization
SBS	Single Base Substitution
SNP	Single Nucleotide Polymorphism
TCGA	The Cancer Genome Atlas
TMB	Tumor Mutational Burden
UCEC	Uterine Corpus Endometrial Carcinoma
VCF	Variant Call Format
ZINB	Zero-Inflated Negative Binomial
