# Supplementary material for: The Tumor Multi-Omic Landscape of Endometrial Cancers Developed on a Background of Adiposity
Source: Genes (Basel). 2026 Jun 29;17(7):744. doi: 10.3390/genes17070744 (PMC13408957; doi:10.3390/genes17070744)
Supplement: Supplementary file 1 [file genes-17-00744-s001.zip › supp-genes-4371151-revised-round-2/SUPP FIGS + CAPTIONS Richenberg et al BMI TCGA UCEC ms 260626.pdf]

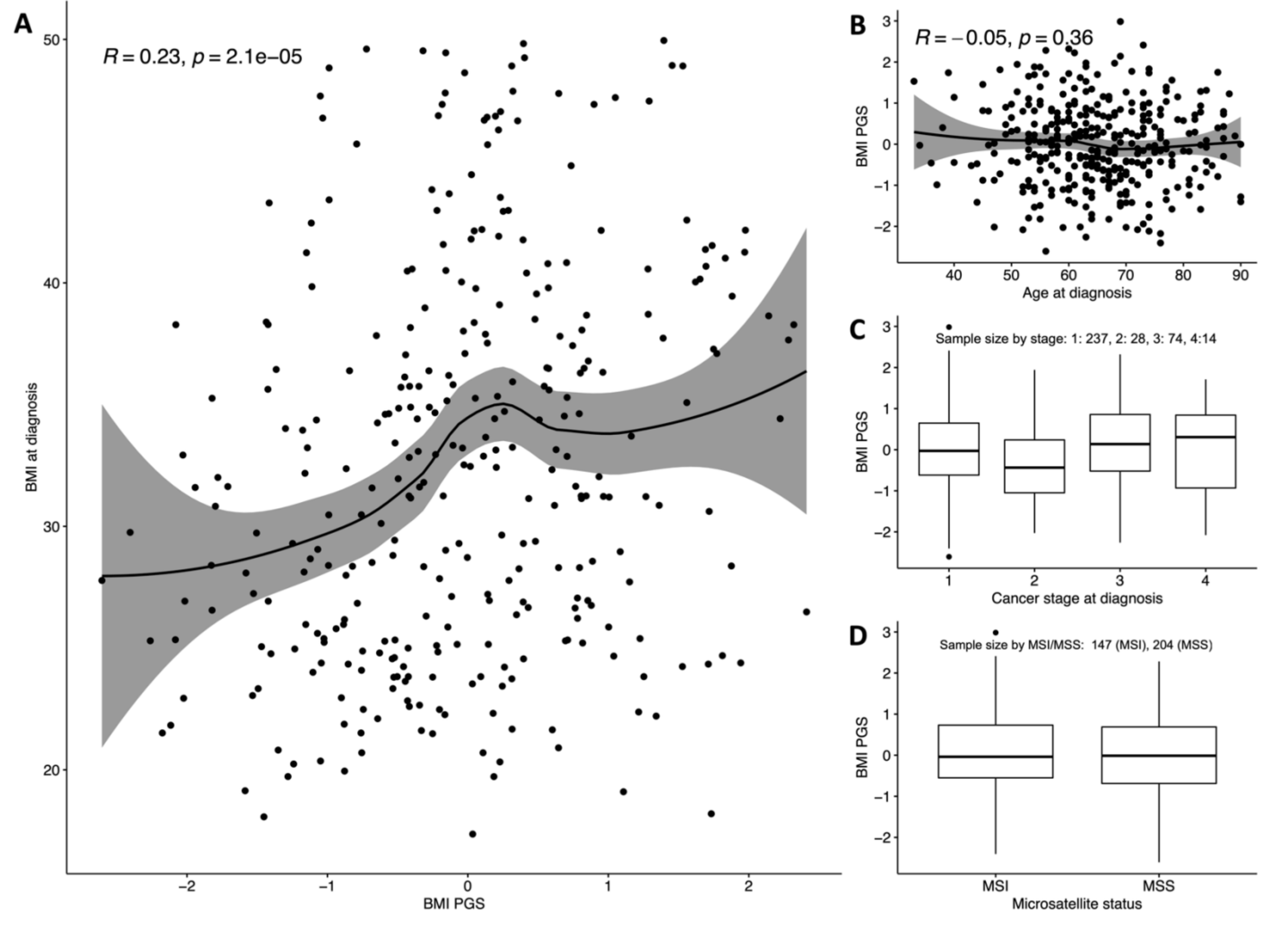

Figure S1: Associations between the BMI germline PGS in standard deviation units and (A) BMI measured at diagnosis (Pearson correlation coefficient; N = 322), (B) age at diagnosis (Pearson correlation coefficient; N = 354), (C) stage at diagnosis (N = 354; simple linear regression beta = 0.05 / P = 0.39), and (D) tumor microsatellite status (N = 351; simple linear regression beta = 0.04 / P = 0.72). The scatter plots in (A) and (B) include the LOESS (locally weighted smoothing) line with 95% confidence interval shaded.

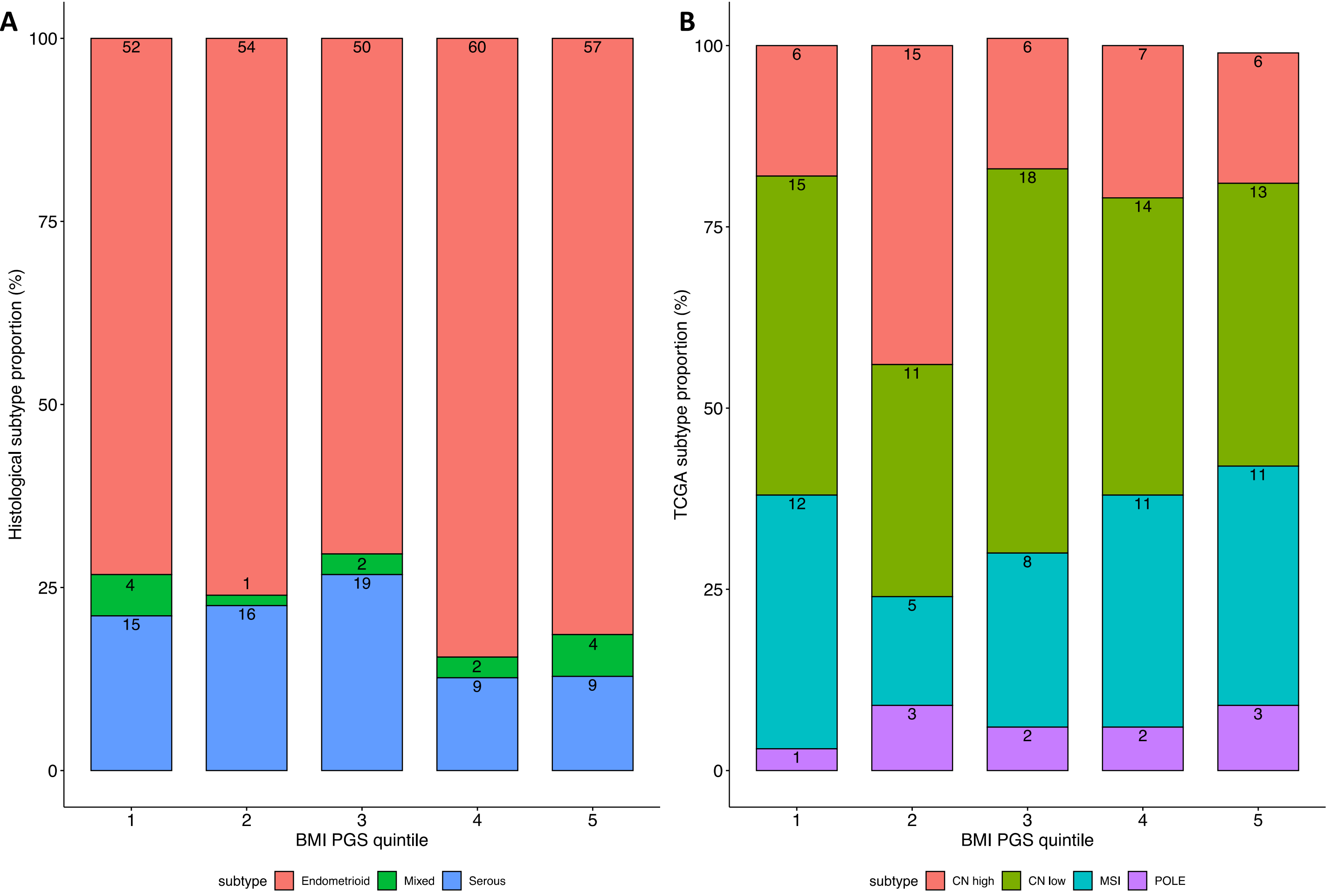

Figure S2: Distribution of endometrial cancer histological (A) and TCGA molecular (B) subtypes by BMI germline PGS quintiles in the TCGA UCEC cohort.
